# Regular, low-dose methadone for reducing breathlessness in people experiencing or at risk of neurotoxic effects from morphine: A single-center case series

**DOI:** 10.3389/fmed.2022.925787

**Published:** 2022-12-05

**Authors:** Piotr Z. Sobanski, David C. Currow

**Affiliations:** ^1^Palliative Care Unit and Competence Centre, Department of Internal Disease, Schwyz Hospital, Schwyz, Switzerland; ^2^Faculty of Science, Medicine and Health, University of Wollongong, Wollongong, NSW, Australia

**Keywords:** breathlessness, opioids, palliative management, heart failure, morphine, methadone

## Abstract

Breathlessness is a common symptom suffered by people living with advanced malignant and non-malignant diseases, one which significantly limits their quality of life. If it emerges at minimal exertion, despite the maximal, guidelines-directed, disease-specific therapies, it should be considered persistent and obliges clinicians to prescribe symptomatic, non-pharmacological, and pharmacological treatment to alleviate it. Opioids are recommended for the symptomatic treatment of persistent breathlessness, with morphine most extensively studied for this indication. It is extensively metabolized in the liver into water-soluble 3- and 6-glucuronides, excreted by the kidneys. In the case of advanced renal failure, the glucuronides accumulate, mainly responsible for toxicity 3-glucuronides. Some people, predominantly those with advanced renal failure, develop neurotoxic effects after chronic morphine, even when prescribed at a very low dose. A single-center case series of consecutive patients experiencing neurotoxic effects after long-term, low-dose morphine or at risk of such effects were transferred to methadone to avoid the accumulation of neurotoxic metabolites. Over the course of 4.5 years, 26 patients have been treated with methadone in the median dose of 3.0 mg/24 h p.o., for persisting breathlessness. Sixteen of them had been treated previously with an opioid (usually morphine) at the median dose of 7.0 mg/24 h (morphine oral daily dose equivalent). They were transferred to methadone, with the median dose of 3.0 mg/24 h orally (methadone oral daily dose equivalent), and the median morphine-to-methadone dose ratio was 2.5:1. All patients experienced a meaningful improvement in breathlessness intensity after methadone, by a median of 5 points (range 1–8) on the 0–10 numerical rating scale (NRS) in the whole group, and by 2 points (range 0–8) in those pretreated with other opioids, mainly morphine. Low-dose methadone can be considered an efficient alternative to morphine for reducing breathlessness in people experiencing neurotoxic effects or at risk of developing them following treatment with morphine.

## Introduction

Breathlessness is a common symptom in advanced malignant and non-malignant life-limiting illnesses. It affects up to 98% of people living with advanced chronic obstructive pulmonary disease (COPD), 88% of those living with advanced heart failure (HF), and 77% of people with advanced cancer (1, 2). In HF, it is so ubiquitous that is considered its hallmark symptom (2, 3). The threshold of exercise evoking breathlessness decreases in parallel to the severity of HF. In the most advanced stages, breathlessness is present with minimal exertion (like speaking, eating, or toileting) or even at rest. It is depicted in the most used classification of staging HF, the New York Heart Association (NYHA) classification. Similarly in COPD, breathlessness is one of the main factors determining its progression accordingly to the Global Initiative for Chronic Obstructive Lung Disease (GOLD) classification (4, 5).

When recognizing new or worsening breathlessness, actively seeking condition(s) that evoke or worsen, it is mandatory (6). Reversible conditions need to be sought actively and treated as effectively as possible. If severe breathlessness persists despite optimal treatment of all underlying conditions, symptomatic non-pharmacological and pharmacological management should be considered (7, 8).

Opioids are the recommended pharmacological therapy for the symptomatic reduction of persisting breathlessness in cancer (9), COPD (10), and HF (3, 11). Morphine is the most studied opioid; hydromorphone, diamorphine, codeine, fentanyl, and buprenorphine have been studied much less frequently for the symptomatic reduction of breathlessness. Fear of potential side effects of opioids (non-respiratory: drowsiness, sedation, impaired cognition, nausea, constipation, immunosuppression, and suppression of testosterone) and respiratory effects (respiratory depression and respiratory failure) limit their prescription, even if they are clinically indicated. Most participants in controlled clinical trials have been prescribed sustained-release morphine. Persisting breathlessness is a registered indication in Australia for sustained-release morphine commencing at 10 mg/24 h with the ability to titrate this by 10 mg/24 h weekly up to a maximum 30 mg/24 h. In most countries, the lowest possible sustained-release morphine dose is 20 mg/24 h (12–14).

The limitation in prescribing morphine is the risk of accumulation, primarily its neurotoxic metabolite [morphine-3-glucuronide (M3G)] in the case of renal insufficiency (15, 16). Regular morphine should be used more cautiously if the glomerular filtration rate is very low (17, 18). Hydromorphone, commonly suggested as an alternative to morphine in this situation, undergoes similar metabolism to morphine [to hydromorphone-3-glucuronide (H3G)], which can accumulate in severe renal impairment, encountering the same risk of neurotoxicity as M3G, and thus should be used with increased caution (16, 17, 19). The risk of neurotoxicity from opioids in people with advanced conditions is probably more common than identified, as impaired renal function is commonly seen in people with advanced disease, especially in advanced HF (20, 21). For this reason, clinicians prescribing opioids should be aware of the possibility of neurotoxic side effects, even if the doses are low. Fentanyl is sometimes suggested for breathlessness management, but the effect of transmucosal fentanyl is very short (about 1 h), making it unsuitable for the management of chronic breathlessness. In this consecutive case series, a bedside decision was made to prescribe methadone for those patients suffering from chronic breathlessness.

Methadone, in the case of renal failure, also accumulates but does not have neurotoxic metabolites, so reduction of doses (to 50–75%), prolonging the intervals between doses, and careful monitoring of effects should be sufficient to prevent overdose (22). Methadone can be prescribed by oral, sublingual, intravenous, subcutaneous, intramuscular, or rectal routes. In people who cannot swallow, sublingual methadone can be especially useful, with a quick onset of action (23).

The aim of this consecutive case series was to describe the effect of methadone on persisting breathlessness in people experiencing unacceptable side effects, or at risk, from other opioids mainly morphine.

## Methods

This article presents a retrospective analysis of medical records of consecutive patients hospitalized in a single palliative care unit in a district hospital in Switzerland, who experienced neurotoxic effects after small doses of morphine prescribed for breathlessness management, due to severe renal failure (stage 4 or 5 according to Kidney Disease Improving Global Outcomes (KDIGO) classification) or at risk of such effects. After the indications for symptomatic breathlessness management had been verified, all other opioids were discontinued (if a patient was not opioid naïve), and the intensity of breathlessness was regularly assessed. Patients who were able to take medicines orally received 1 mg methadone s.l., and then twice daily if needed. If the patient experienced a satisfactory reduction in breathlessness, the planned dose was delayed or omitted. If needed, an additional dose was allowed at least 3 h after the last methadone dose. After 3–4 days, the total daily dose was calculated, based on the mean daily dose in the previous 48 h, and usually prescribed every 12 h.

## Results

From September 2017 to February 2022, 26 consecutive patients were included (14 women and 12 men), median age of 73.5 years (range 55–94). Eighteen patients had cancer, seven had advanced chronic obstructive pulmonary disease (COPD) with a median GOLD score of 3 (of whom four also had cancer), two people predominantly had heart failure, and two people had amyotrophic lateral sclerosis (ALS). Fifteen people used oxygen therapy through nasal prongs (median 2 L/min), with a median pulse oximetry of 94%, generating similar measures to patients not on oxygen (94%). Patients with ALS were treated with non-invasive positive pressure ventilation (servo-ventilation), one during the night hours only, the other progressively until continuous ventilation was required in the time directly preceding her death. The median glomerular filtration rate (GFR) in the whole group was 61 ml/min/1.73 m^2^ body surface area (BSA), but in 5 people the GFR was under 30 ml/min/1.73 m^2^ BSA (range 16–27). The detailed patient characteristics are depicted in Supplementary Table 1.

All patients were informed that methadone treatment for the symptomatic reduction of persisting breathlessness is not standard therapy. All agreed to try this approach; however, one person [ultimately the person with the longest period of opioid treatment (550 days)] was originally hesitant to start taking opioids, as she did not consider herself as dying. Eighteen patients died during hospitalization, one was discharged and seen in an ambulatory capacity (this person, under supervision of the general practitioner, gradually lowered the dose and finally discontinued methadone after 550 days of treatment, due to the improvement of exercise tolerance after breathlessness had been alleviated), and seven patients were discharged from the palliative care unit alive and not followed up as the study was a retrospective analysis of medical records, not a clinical study.

### Breathlessness assessment

The breathless burden was assessed using the modified Medical Research Council (mMRC) classification and the intensity using a 0–10 Numerical Rating Scale (NRS). Before starting the opioids, the breathlessness median grade was 4 according to mMRC (range 2–4; two people with grade 2; nine with grade 3, and 14 with grade 4), the median intensity was 8 NRS (range 5–10). One person experiencing breathlessness at rest was unable to describe its intensity and in the records of another person, mMRC and NRS assessments were missing with only the narrative information about the improvement of breathlessness documented.

Sixteen of the 26 people had already been on an opioid for persisting breathlessness: (morphine—14 people, morphine then oxycodone—1 person, and hydromorphone—1 person).

All included in this analysis reported improvements under methadone in breathlessness—by mMRC grade median from 4 (range 2–4) to median 3 (range 2–4), and the median difference was 1 (range 0–3). NRS scores shifted from a median 8 (range 5–10) to 3 (range 2–7), with a median difference of 5 (range 1–8) vs. pretreatment.

After starting methadone (9 people methadone was prescribed *de novo* and 1 person received only one rescue dose of morphine and was perceived as opioid naive), the median mMRC grade was 3 (range 2–4) in the whole population, 2 (range 2–4) in those who had been pretreated with another opioid, and 3 (range 3–4) in those who were put directly on methadone. The median NRS when prescribed methadone was 3 (range 1–6) in all groups.

The median time on methadone was 21 days (range 3–550).

Two to three days after starting methadone, two people reported breathlessness at rest (mMRC grade 4), 11—mMRC grade 3, and 10—mMRC grade 2. For one patient, breathlessness was not experienced at rest, but more detailed grading was not possible as he was completely bedbound, and in the case of two others, only descriptive data were available from the notes.

### Dosing of opioids

The median dose when pre-treated with opioids expressed as morphine oral daily dose equivalent was 7 mg/(range 2.5–23). The median steady-state methadone oral daily dose was 3 mg (range 0.5–9.5). The median dose ratio between morphine and methadone after reaching stable improvement (72 h after final dose adjustment) was 2.5 (interquartile range 1.9–4, and data of one patient transferred from a very high-dose hydromorphone prescribed predominantly for pain management, calculated as morphine oral daily dose equivalence 180 mg has been omitted from all calculations).

### Illustrative cases

Patient #1 developed hallucinations and drowsiness that lasted about 36 h after a single dose of 5 mg intermediate-release (IR) oxycodone. The breathlessness alleviation was shorter than the duration of hallucination (about18–24 h). After hospital admission, oral oxycodone was rotated to morphine and administered subcutaneously in a syringe driver starting with 5 mg/24 h without any neurotoxic effects but with some improvement in breathlessness. The dose needed to be escalated gradually to 10 mg s.c./24 h. With this dose, drowsiness (but not hallucinations) became evident. The patient was rotated to an i.v. infusion of methadone, and the dose was gradually titrated to 5 mg/24 h (calculated as 6 mg methadone oral daily dose equivalent) with significant improvement of consciousness and satisfactory breathlessness improvement. To optimize symptom control, the dose was gradually increased to 8 mg i.v. (oral dose equivalence 9.5 mg) without causing relevant sedation or hallucinations.

Patient #6 with breathlessness caused by alveolar hypoventilation secondary to ALS had occasionally tried in the past to use 1 mg intermediate-release morphine orally up to maximum of 3 times per day. It always changed the character of breathlessness at rest from air hunger to qualitatively very different thoracic and neck rigidity disturbing breathing, being a little less distressing than the original breathlessness. Starting 1 mg methadone, once daily orally, alleviated the breathlessness successfully without causing the unpleasant effect of muscle stiffness. The effect remained stable for several months. Occasionally, the patient requested a pause in the administration of methadone, to make sure its continuation was still needed. Breathlessness relapsed every time within 2–3 days and settled as methadone was restarted. This pattern lasted for several months until death.

Patient #8 had oxygen-dependent, severe COPD with persisting breathlessness, which was well-controlled for 7 months on morphine 8 mg/24 h (oral IR solution—2 mg every 6 h). The person developed hallucinations (the cause of an emergency room presentation) likely caused by morphine metabolites accumulation secondary to acute renal failure. After rehydration and discontinuation of morphine, the hallucinations disappeared, but breathlessness reappeared after 18–24 h. Because the breathlessness reappeared before the normalization of renal function and the patient declined continued morphine (due to the experience of the hallucinations), oral methadone was commenced (1 mg every 12 h) providing good relief from breathlessness, with the benefit lasting more than 10 months at that dose. With methadone, the patient was able to gradually increase her exercise capacity while still relying on around-the-clock oxygen therapy and walking aids.

Patient #20 with lung cancer and a malignant pleural effusion treated with drainage reported increasing tiredness preventing dose escalation after starting morphine IR 2 mg every 6 h. Breathlessness NRS scores had dropped from 7 to 4. The introduction of 2 mg methadone per day (escalated to 3 mg) generated quantitative and qualitative improvements in breathlessness. Two weeks after methadone was introduced, hospital admission was required due to the worsening of breathlessness and deterioration of the general condition. He died 1 week after breathlessness alleviation was reached with a methadone dose escalation to 10 mg.

## Discussion

Breathlessness is a common symptom in people living with advanced diseases, both in malignant and non-malignant life-limiting illnesses. If it remains severe despite optimal disease-specific treatment, it should be considered “persisting” and oblige clinicians to initiate symptomatic management, consisting of non-pharmacological interventions and pharmacological treatment mainly by regular, low-dose opioids, particularly morphine (9, 12, 14). Morphine is extensively and rapidly metabolized in the liver, especially after oral administration, to morphine-6-glucuronide (M6G) considered as main metabolite responsible for pharmacologically desired effects, and M3G perceived as probably responsible for adverse, neurotoxic effects. M3G is more likely to accumulate in severe renal failure. For this reason, opioids that do not have potentially harmful metabolites that can be used in this population are relevant. In this consecutive case series of 26 patients who experienced or were at risk of neurotoxicity from other opioids, all were successfully treated with methadone in a single Swiss center over a 4.5-year course.

All treated patients experienced clinically meaningful improvements in breathlessness intensity after methadone administration by a median of 5 (range 1–8) in the whole group in comparison to pretreatment (change from median 8 to median 3 points) on the NRS. Those who were pretreated with another opioid experienced further reductions in breathlessness in comparison to change after morphine by a median of 2 (range 0–8; from median to median 2.5) points on the NRS. The median improvement of breathlessness after morphine in comparison to pretreatment was 3 points on the NRS (range 0–7 points). According to mMRC after starting methadone, the median improvement reached 1 point (range 0–3) for methadone vs. pre-treatment in the whole group, 1 point (range 0–2) vs. pretreatment in those directly commenced on methadone, and further reductions in breathlessness in comparison to change under morphine by 1 point (range 0–2). The current study outlines the improvement of breathlessness after methadone even in people who had experienced symptomatic benefits from a previous opioid (10, 12, 14). The absorption of methadone from oral mucosa allowed convenient administration of single drops, even in unconscious patients. No one reported neurotoxic effects. One person required a dose reduction to 0.5 mg methadone daily due to slight sedation.

The median methadone oral daily dose equivalence needed for stable symptom management was 3 mg (range 0.5–9.5). For those who were previously treated with other opioids (*n* = 16), mainly morphine, the median morphine oral daily dose equivalence was 7 mg. The median of the dose ratio between morphine and methadone was 2.5:1 (interquartile range 1.9–4), a little less than described by other authors (24). It can be hypothesized that patients in our study were treated with a little higher than equipotent dose of opioids. Despite this, they reported better management of breathlessness from methadone, both quantitatively and qualitatively, including a few patients who ceased taking morphine and commenced course methadone. That could mean slightly different receptor affinities of opioids are targeted.

### Limitations

This is a retrospective record analysis of patients treated in a single center. All non-randomized and non-blinded studies bear considerable risks of bias, and any causative relationship of pharmacological interventions with clinical outcomes needs to be interpreted with caution.

## Conclusion

Low-dose methadone can be considered as an alternative to other opioids, for breathlessness management, in those who experience neurotoxic effects after other opioids, or those at risk of such effects (due to *severe* renal failure), although this hypothesis requires further exploration. Variations in its half-life make it more challenging to titrate, and this needs to be done on a case-by-case basis. Further studies are needed to verify these preliminary findings.

## Data availability statement

The raw data supporting the conclusions of this article will be made available by the authors, without undue reservation.

## Ethics statement

Ethical review and approval was not required for the study on human participants in accordance with the local legislation and institutional requirements. Written informed consent for participation was not required for this study in accordance with the national legislation and the institutional requirements.

## Author contributions

PS chaired the Palliative Care Department, where the case series has been recruited. The design of the retrospective analysis, a draft of the manuscript, presentation of data, and interpretation of data have been developed by both authors. Both authors agreed the final version of the manuscript.
